# Sensitization to Cor a 9 and Cor a 14 is highly specific for a severe hazelnut allergy in Dutch children and adults

**DOI:** 10.1186/2045-7022-3-S3-O14

**Published:** 2013-07-25

**Authors:** L Masthoff, L Mattsson, L Zuidmeer-Jongejan, J Lidholm, K Andersson, JH Akkerdaas, SA Versteeg, C Garino, Y Meijer, P Kentie, A Versluis, CF den Hartog Jager, CA Bruijnzeel-Koomen, AC Knulst, R van Ree, E van Hoffen, SG Pasmans

**Affiliations:** 1 Dermatology/Allergology, University Medical Center Utrecht, Utrecht, the Netherlands; 2 Thermo Fisher Scientific, Uppsala, Sweden; 3 Experimental Immunology, Academic Medical Center, Amsterdam, the Netherlands; 4 Pharmaceutical Sciences, Drug & Food Biotechnology Center, University of Piemonte Orientale "A. Avogadro", Novara, Italy; 5 Pediatric Pulmonology/Allergology, University Medical Center Utrecht, Utrecht, the Netherlands

## Background

Component-resolved diagnosis has been shown to improve diagnosis of food allergy. The aim of this study was to evaluate whether component-resolved diagnosis may help to identify patients at risk of severe allergic reactions to hazelnut.

## Methods

A total of 161 hazelnut-sensitized patients were included: 40 children and 15 adults with objective symptoms in DBPCFC and 24 adults with a convincing severe history were compared to 41 children and 41 adults with no or subjective symptoms in DBPCFC (grouped together). IgE levels to hazelnut extract and single components were analyzed with ImmunoCAP.

## Results

IgE to hazelnut extract was significantly higher in children with severe than with no or mild symptoms. Sensitization to rCor a 1.04 was common among both children and adults, while sensitization to rCor a 8 was rare. In 13% of children and 49% of adults with a severe hazelnut allergy, only sensitization to rCor a 1.04 was observed and not to other water-soluble allergens. Sensitization to nCor a 9 and/or rCor a 14 was strongly associated with a severe hazelnut allergy. Using adapted cut-off levels, a diagnostic discrimination between severity groups was obtained. IgE to either nCor a 9 ≥1 kU_A_/L or rCor a 14 ≥5 kU_A_/L (children) and IgE to either nCor a 9 ≥1 kU_A_/L or rCor a 14 ≥1 kU_A_/L (adults) had a specificity of >90% and accounted for 83% of children and 44% of adults with a severe hazelnut allergy.

## Conclusion

Sensitization to Cor a 9 and Cor a 14 is highly specific for patients with objective symptoms in DBPCFC as marker for a more severe hazelnut allergic phenotype.

## Disclosure of interest

None declared.

